# Is calprotectin a reliable marker in surgical ICU settings? A clinical evaluation of its role in sepsis and mortality prediction

**DOI:** 10.3389/fmed.2025.1619825

**Published:** 2025-10-10

**Authors:** Ebru Aladağ, Muhammed Emin Zora, Yücel Gültekin, Soycan Mızrak

**Affiliations:** ^1^Department of Anesthesiology and Reanimation, Faculty of Medicine, Alanya Alaaddin Keykubat University, Alanya, Türkiye; ^2^Department of Anesthesiology and Reanimation, Faculty of Medicine, Uşak University, Uşak, Türkiye; ^3^Intensive Care Unit, Department of General Surgery, Faculty of Medicine, Aydın Adnan Menderes University, Aydın, Türkiye; ^4^Department of Medical Biochemistry, Faculty of Medicine, Uşak University, Uşak, Türkiye

**Keywords:** biomarkers, calprotectin, mortality prediction, sepsis, surgical intensive care unit

## Abstract

**Background:**

Calprotectin, a neutrophil-derived protein, has emerged as a potential biomarker for inflammation and infection. This study evaluated the utility of serum calprotectin levels in diagnosing sepsis and predicting mortality in surgical intensive care unit (ICU) patients.

**Methods:**

This prospective observational study included 124 patients admitted to the surgical ICU at Uşak Training and Research Hospital between 2022 and 2024. Data on demographics, serum calprotectin, CRP, PCT, lactate levels, and clinical scores (SOFA, APACHE II) were collected. ROC analysis was used for predictive accuracy; Spearman and Pearson correlation coefficients assessed associations.

**Results:**

No significant associations were found between serum calprotectin and SOFA/APACHE II scores, CRP, or lactate. However, calprotectin correlated positively with PCT in sepsis (*r* = 0.428, *p* = 0.002) negatively in postoperative subgroups (*r* = −0.48, *p* < 0.001). Calprotectin showed poor prognostic accuracy (AUC = 0.472). Mortality was significantly associated with high CRP and severity scores, but not with calprotectin levels.

**Conclusion:**

Calprotectin alone lacks sufficient diagnostic or prognostic power for sepsis in surgical ICU patients. It may serve as a complementary marker alongside PCT in specific subgroups.

## Introduction

Sepsis is a severe clinical condition characterized by life-threatening organ dysfunction resulting from a dysregulated host response to infection (1). Global Burden of Disease studies indicate that 19.7% of all deaths worldwide are attributable to sepsis (2). Early diagnosis and treatment of sepsis are critical to reducing mortality. However, diagnosing sepsis can be clinically challenging, as infections that initially present with mild symptoms may rapidly progress to life-threatening organ dysfunction or septic shock. Therefore, reliable biomarkers are needed alongside clinical expertise to assist clinicians in diagnosis and risk stratification.

Calprotectin is a calcium-binding protein with a heterodimeric structure composed of the S100A8 and S100A9 proteins, accounting for approximately 60% of the soluble protein content in the cytosol of neutrophilic leukocytes. Released in large quantities during neutrophil activation or cell death, calprotectin functions as a potent alarmin, initiating and sustaining inflammatory processes, and in murine models, it has been shown to activate Toll-like receptor 4 and promote lethal endotoxin-induced shock (3). Calprotectin, a routinely used marker for the presence of neutrophils in stool in inflammatory bowel disease, has also attracted attention as a potential biomarker for acute infection and sepsis, as it is released early during the host response to infection. Blood levels have therefore been investigated as a possible early marker, possibly rising within hours of onset (4). Furthermore, calprotectin has been investigated as a potential aid in the differentiation between viral and bacterial infections (4–6).

Recent studies have demonstrated that calprotectin levels are significantly elevated in patients who develop sepsis and are associated with disease severity. High calprotectin levels in severe infection have been investigated as markers of disease severity, although no consistent correlation has been demonstrated (7). Several studies have reported that serum calprotectin levels in patients diagnosed with sepsis are significantly higher compared to control groups and that baseline calprotectin concentrations are associated with mortality risk (8). More recent comparative studies conducted in intensive care units and emergency departments have shown that calprotectin exhibits markedly superior diagnostic accuracy for sepsis compared to procalcitonin (PCT) and has been found to outperform PCT in predicting 30-day mortality (9).

This study aimed to investigate the diagnostic and prognostic value of serum calprotectin in surgical ICU patients with suspected sepsis and to compare its performance with that of established biomarkers, such as procalcitonin. We also examined whether calprotectin levels at admission were associated with short-term mortality.

## Materials and methods

### Study design and participants

**Figure 1:**
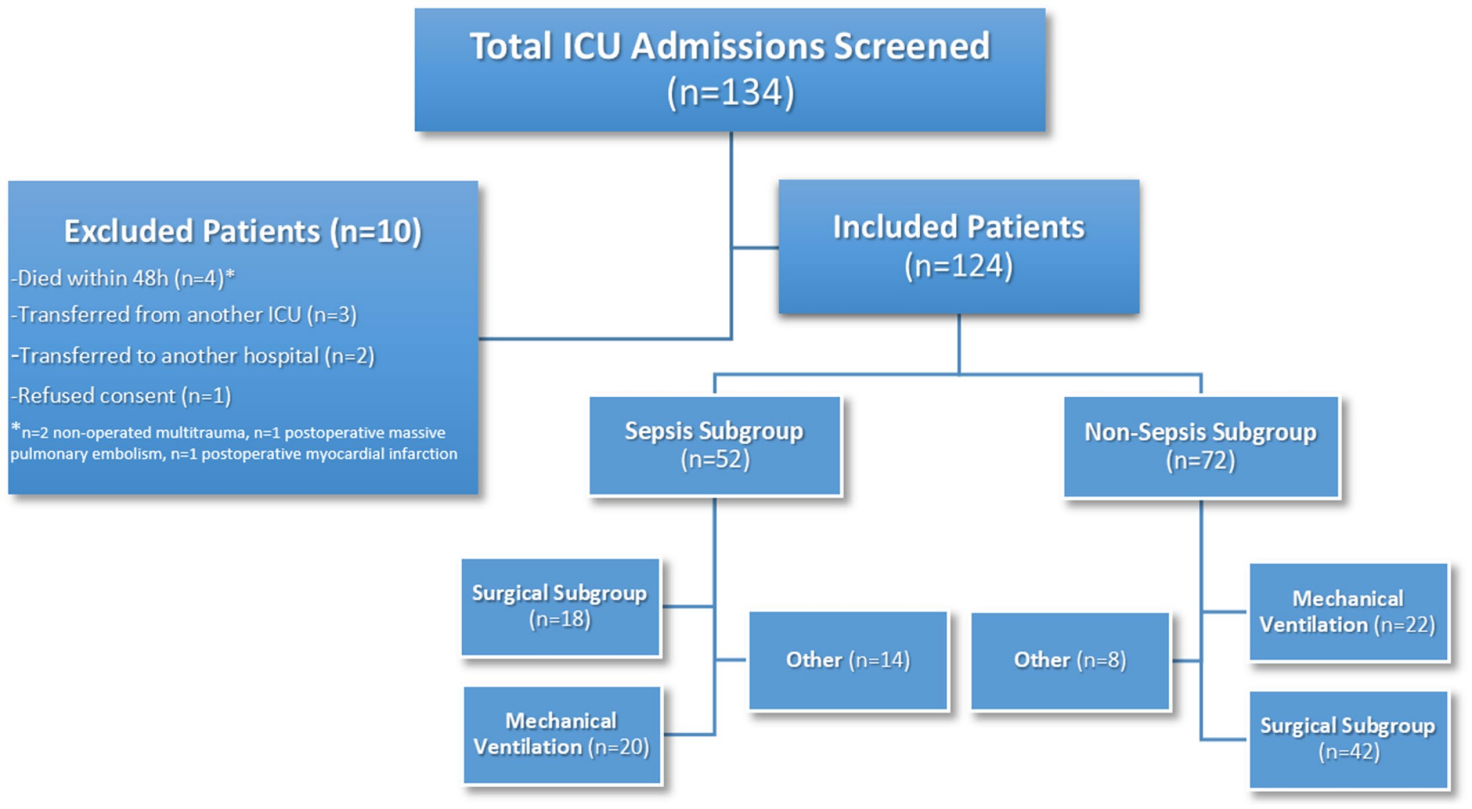
Flow diagram of patient selection and subgroup classification in the surgical ICU cohort.

This prospective observational study was conducted between 2022 and 2024 in the Surgical Intensive Care Unit of Uşak Training and Research Hospital. A total of 134 ICU patients were screened. Based on predefined exclusion criteria, 10 patients were excluded, and the final analysis included 124 patients (Figure 1).

To minimize potential confounding factors that could influence calprotectin levels, patients with known active malignancy or chronic inflammatory or autoimmune diseases (e.g., rheumatoid arthritis, inflammatory bowel disease) were excluded from the study. These conditions were identified through clinical evaluation, medical history, and diagnostic records at the time of ICU admission.

In addition, patients admitted primarily for observation or with limited intensive care needs (e.g., postoperative monitoring after minor surgical procedures), as well as those who experienced early discharge or rapid clinical deterioration due to complications such as hemodynamic instability or postoperative bleeding, were excluded from the study. Calprotectin samples were not collected from these patient groups.

To ensure the inclusion of patients with genuine intensive care needs and to avoid enrolling those admitted for short-term monitoring or with low-acuity conditions, patients with an ICU length of stay of less than 3 days were not included. Furthermore, patients who died within the first 48 h of ICU admission were excluded from the final analysis, even if calprotectin samples had been obtained. These early deaths were primarily attributable to postoperative complications and hemodynamic instability rather than sepsis.

#### Infection management

In accordance with the Sepsis-3 guidelines, all patients diagnosed with sepsis or septic shock received empiric broad-spectrum antibiotic therapy upon diagnosis. Antimicrobial treatments were subsequently adjusted based on microbiological culture results and clinical response.

#### Inclusion criteria

- Age ≥18 years.
- Provision of informed consent by the patient or a legal representative.
- A minimum ICU stay of 3 days.

#### Exclusion criteria

- Age under 18 years.
- Absence of informed consent.
- Transfer from another ICU or transfer to another facility during ICU follow-up.
- Death within the first 48 h following ICU admission.
- ICU stay of less than 3 days.
- Known active malignancy.
- Diagnosed chronic inflammatory or autoimmune diseases (e.g., rheumatoid arthritis, inflammatory bowel disease).
- Patients with limited ICU needs or admitted for observation.

### Data collection and definitions

At the 24th hour of ICU admission, venous blood samples were obtained from all patients to measure serum levels of procalcitonin (PCT), C-reactive protein (CRP), lactate, and calprotectin. Additionally, both the Acute Physiology and Chronic Health Evaluation II (APACHE II) and the Sequential Organ Failure Assessment (SOFA) scores were calculated at the same time point to assess illness severity and organ dysfunction, respectively. Biomarker sampling was standardized at the 24th hour to minimize variability caused by immediate postoperative stress or resuscitation procedures and to reflect the stabilized inflammatory response, as previously described in similar ICU biomarker studies (10).

Sepsis was defined in accordance with the Sepsis-3 criteria as life-threatening organ dysfunction resulting from a confirmed or suspected infection, indicated by an increase of ≥2 points in the SOFA score. In this study, the baseline SOFA score was assumed to be zero in patients without known pre-existing organ dysfunction, and sepsis was diagnosed when the SOFA score at ICU admission was 2 or greater, consistent with approaches used in previous ICU-based biomarker studies (10).

Baseline demographic and clinical characteristics, including age, sex, comorbidities, and Charlson comorbidity index (CCI) scores, were extracted from the hospital’s electronic medical records and ICU admission notes at the time of enrollment.

Patients were followed until ICU discharge or in-ICU death. Mortality data reflect ICU mortality only; no post-discharge or long-term follow-up was conducted.

### Calprotectin measurement

Serum calprotectin levels were measured using a commercial ELISA kit (SunRed Bio, Shanghai, China) following the manufacturer’s instructions. The assay range was 0.15–40 ng/mL, with intra-assay and inter-assay coefficients of variation of less than 10 and 12%, respectively.

### Sample quality control

To ensure the integrity and reliability of serum samples before analysis, standard quality control measures commonly accepted in clinical research were followed during the sampling and storage processes. Venous blood samples were collected under sterile conditions into additive-free dry tubes and centrifuged within 2 h of collection at 3,000 rpm for 20 min to obtain serum. The serum samples were stored at −80 °C to preserve biomarker stability. During ELISA analysis, standard curves and control samples provided by the manufacturer were used according to the recommended protocol. The reported intra-assay and inter-assay coefficients of variation for the assay kit were below 10 and 12%, respectively. All measurements were conducted within the manufacturer’s recommended time limits. Serum samples were analyzed within 8 weeks of storage at −80 °C to ensure biomarker stability, in line with published pre-analytical data on calprotectin stability.

### Sample size and power analysis

**Figure 2:**
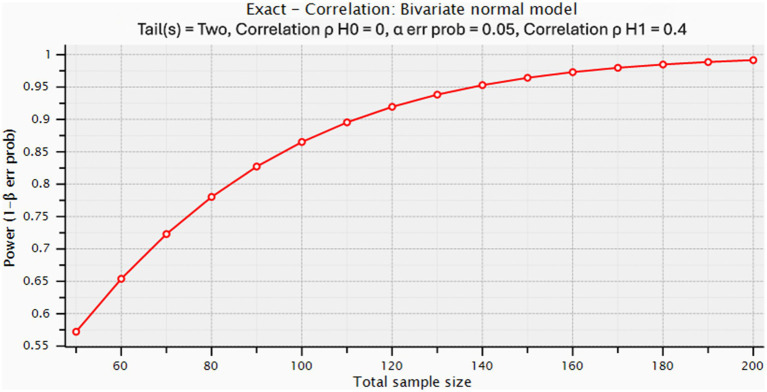
Statistical power analysis for correlation between calprotectin and other biomarkers.

At the beginning of the study, a statistical power analysis was conducted to determine the target sample size. Calculations were performed using G*Power 3.1 software (11). A significance level of *α* = 0.05 and a statistical power (1 − *β*) of 0.90 were assumed. Based on these parameters, a minimum of 121 patients was estimated to be required to detect a moderate correlation (approximately *r* = 0.40) between calprotectin levels and PCT, CRP, and lactate (Figure 2).

Additionally, for the planned ROC (receiver operating characteristic) curve analysis aimed at evaluating the discriminatory performance of calprotectin in predicting mortality, it was assumed that the area under the curve (AUROC) would be approximately 0.60 (moderate level), for which at least 53 patients would be sufficient. Based on these calculations, the study aimed to include at least 124 patients to ensure adequate statistical power, and this target was achieved.

### Statistical analysis

Data analysis was performed using SPSS (Statistical Package for the Social Sciences) version 26.0. The normality of distribution for continuous variables was assessed using the Shapiro–Wilk test. Variables with normal distribution were expressed as mean ± standard deviation, while non-normally distributed variables were presented as median (interquartile range). Categorical data were summarized as counts and percentages (%).

**Figure 3:**
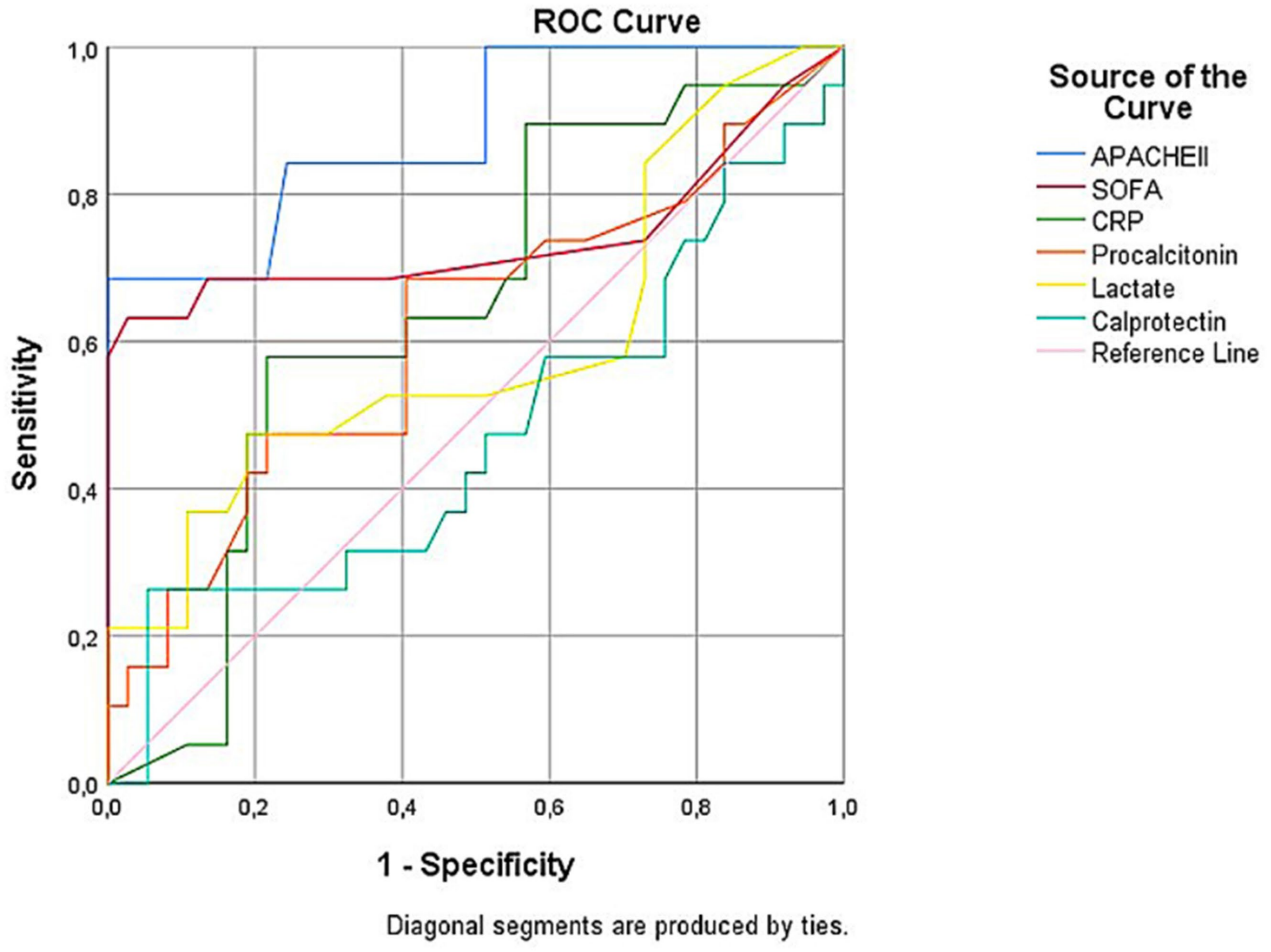
Receiver operating characteristic (ROC) curves comparing the prognostic performance of APACHE II, SOFA, CRP, procalcitonin, lactate, and calprotectin in predicting mortality. APACHE II showed the highest discriminatory ability (AUC = 0.883, 95% CI: 0.815–0.950), followed by SOFA (AUC = 0.740), CRP (AUC = 0.642), procalcitonin (AUC = 0.614), and lactate (AUC = 0.594). Calprotectin demonstrated poor prognostic accuracy (AUC = 0.472). All AUCs, except for lactate and calprotectin, were statistically significant.

The Student’s *t*-test was used for normally distributed variables for comparisons between two groups, while the Mann–Whitney *U* test was applied for non-normally distributed variables. The predictive performance of calprotectin for mortality was evaluated using receiver operating characteristic (ROC) analysis (Figure 3).

Relationships between calprotectin and other clinical parameters were analyzed using Pearson or Spearman correlation coefficients, as appropriate. Finally, multivariate logistic regression analysis was conducted to identify independent mortality-related variables. A *p*-value of <0.05 was considered statistically significant for all tests.

## Results

This study included 124 patients. The mean age was 60.2 ± 20.1 years, with 46.8% being female and 53.2% male. Baseline and clinical characteristics of the study population are summarized in Table 1. Surgical intervention was performed in 48.4% of the patients. The proportion of patients diagnosed with sepsis was 41.9, and 33.9% required mechanical ventilation support. Of the 42 patients who required mechanical ventilation, 20 (47.6%) had sepsis, while 22 (52.4%) were ventilated due to non-septic causes such as postoperative respiratory failure or underlying comorbidities.

**Table 1:** Baseline and clinical characteristics of the study population.

Characteristic	Value
Total number of patients	124
Age (years), mean ± SD	60.2 ± 20.1
Sex (male/female), *n* (%)	66 (53.2%)/58 (46.8%)
Charlson comorbidity index (CCI), median (IQR)	6 (4–9)
Hypertension, *n* (%)	48 (38.7%)
Diabetes mellitus, *n* (%)	34 (27.4%)
Chronic heart disease, *n* (%)	22 (17.7%)
Chronic kidney disease, *n* (%)	13 (10.5%)
Chronic obstructive pulmonary disease, *n* (%)	15 (12.1%)
Dementia/cognitive disorder, *n* (%)	8 (6.5%)
Surgical intervention, *n* (%)	60 (48.4%)
Mechanical ventilation, *n* (%)	42 (33.9%)
Sepsis, *n* (%)	52 (41.9%)
Septic shock, *n* (%)	17 (13.7%)
ICU length of stay, median (IQR)	12 (9–20)
Outcome (survived/died), *n* (%)	86 (69.4%)/38 (30.6%)

CCI, Charlson comorbidity index; IQR, interquartile range. Data are presented as mean ± standard deviation (SD), median (IQR), or number (percentage). Percentages are calculated from the total study population (*n* = 124). ICU mortality refers to deaths during the ICU stay only.

**Figure 4:**
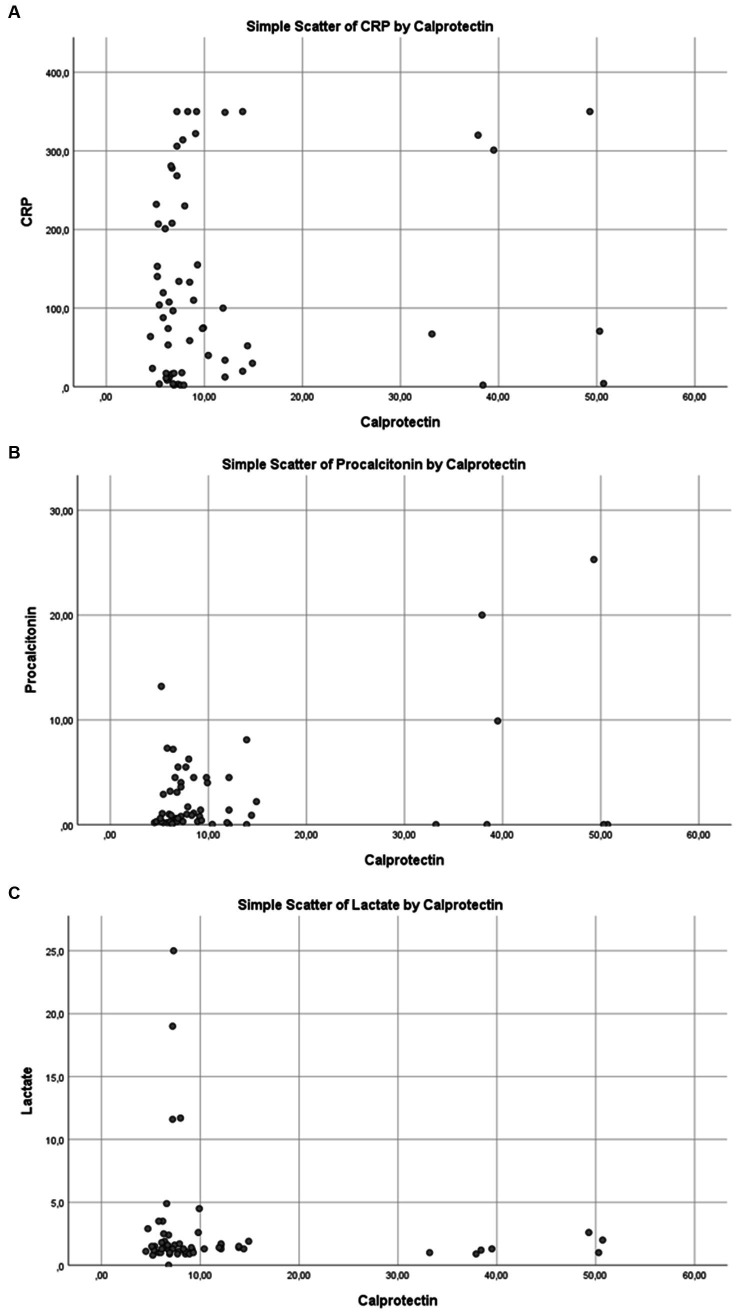
Correlation between serum calprotectin levels and inflammatory/metabolic biomarkers in the overall study population: **(A)** C-reactive protein (CRP), **(B)** procalcitonin, and **(C)** lactate.

No statistically significant correlation was found between serum calprotectin levels and APACHE II or SOFA scores, which are indicators of disease severity. Similarly, in the overall patient population, no significant relationship was observed between calprotectin and CRP, procalcitonin (PCT), or lactate levels (*p* > 0.05; Table 2 and Figures 4A–C).

**Table 2:** Clinical characteristics and correlations with calprotectin levels.

Patient group	Parameters	Median (min–max)	Mortality prediction (*p*-value)	Correlation with calprotectin (*p*-value)	Spearman’s correlation (*r*)
All patients (*n* = 124)	APACHE II	14.5 (1–40)	<0.001	0.13	−0.14
	SOFA	3 (1–15)	<0.001	0.38	0.08
	CRP (mg/L)	98.3 (2–350)	0.002	0.42	0.07
	Procalcitonin (ng/mL)	0.95 (0.01–25.3)	0.003	0.87	0.02
	Lactate (mmol/L)	1.4 (1–19)	0.21	0.43	−0.07
	Calprotectin (ng/mL)	7.4 (4.5–50.7)	0.83	—	1.000
Sepsis subgroup (*n* = 52)	APACHE II	21 (7–40)	<0.001	0.09	−0.24
	SOFA	6 (1–15)	<0.001	0.49	−0.01
	CRP (mg/L)	232 (58.6–350)	0.94	0.18	0.19
	Procalcitonin (ng/mL)	1.1 (0.2–25.3)	<0.001	0.002	0.43
	Lactate (mmol/L)	1.4 (1–19)	0.07	0.73	−0.05
	Calprotectin (ng/mL)	8.3 (5.1–49.3)	0.27	—	1.000
Patients on mechanical ventilation (*n* = 42)	APACHE II	21 (8–40)	<0.001	0.41	−0.13
	SOFA	8 (3–15)	<0.001	0.26	−0.18
	CRP (mg/L)	87.8 (2–306)	0.001	0.006	−0.42
	Procalcitonin (ng/mL)	1.4 (0.01–13.2)	<0.001	0.24	−0.19
	Lactate (mmol/L)	1.7 (0.8–19)	0.07	0.38	−0.14
	Calprotectin (ng/mL)	7.2 (4.7–38.4)	0.84	—	1.000
Surgical subgroup (*n* = 60)	APACHE II	11 (1–21)	0.66	0.06	−0.07
	SOFA	3 (1–9)	0.07	0.06	−0.07
	CRP (mg/L)	79.2 (2–350)	0.22	0.06	0.07
	Procalcitonin (ng/mL)	0.9 (0.01–13.2)	0.38	<0.001	−0.48
	Lactate (mmol/L)	1.4 (0.8–25)	0.01	0.74	−0.05
	Calprotectin (ng/mL)	7.7 (5.2–50.7)	0.93	—	1.000

Data are presented as median (minimum–maximum). Mortality prediction *p*-values were obtained using the Mann–Whitney *U* test to compare survivors and non-survivors. Correlation *p*-values and Spearman’s correlation coefficients indicate the association with calprotectin. APACHE II, Acute Physiology and Chronic Health Evaluation II; SOFA, Sequential Organ Failure Assessment; CRP, C-reactive protein; PCT, procalcitonin.

### Subgroup analyses

**Figure 5:**
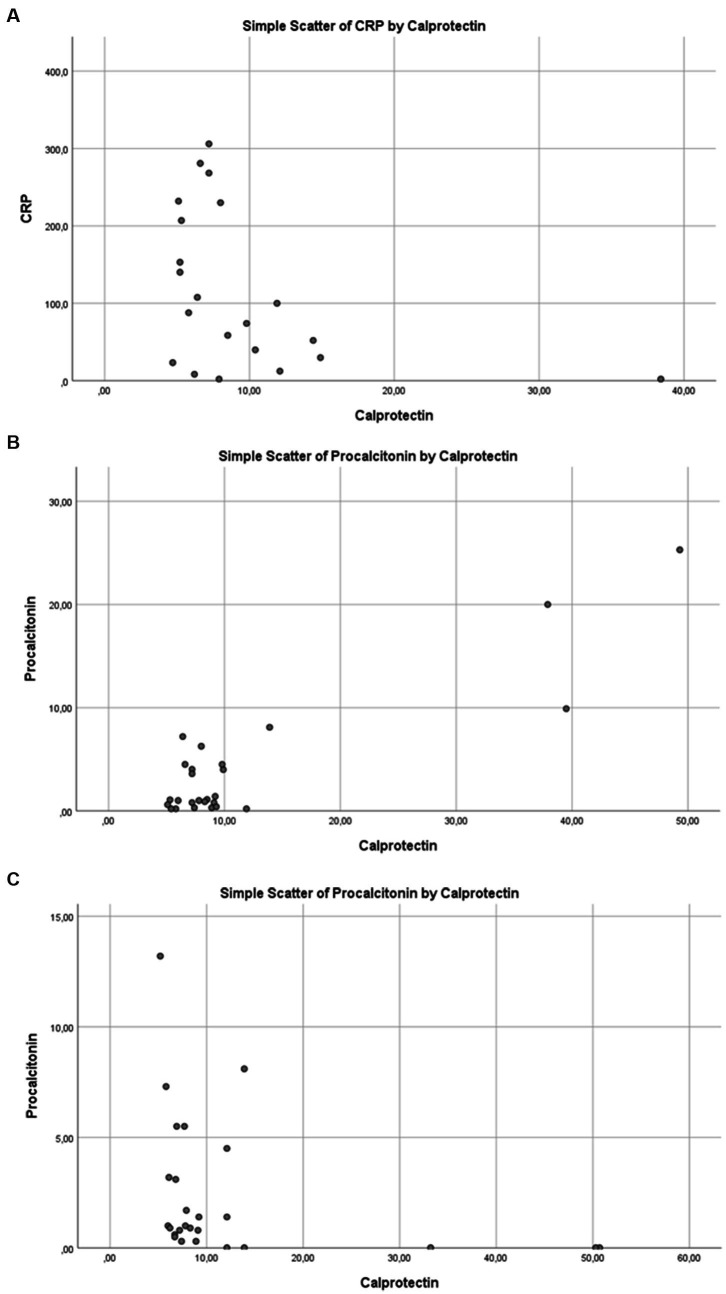
Spearman’s correlation between serum calprotectin levels and selected biomarkers in specific clinical subgroups: **(A)** C-reactive protein (CRP) in mechanically ventilated patients, **(B)** procalcitonin in patients with sepsis, and **(C)** procalcitonin in postoperative intensive care unit (ICU) patients.

In subgroup analyses, a moderate negative correlation was observed between calprotectin and CRP levels in patients requiring mechanical ventilation (Spearman’s rho = −0.42; *p* = 0.006; Figure 5A). Among patients diagnosed with sepsis, a statistically significant moderate positive correlation was found between calprotectin and PCT levels (Spearman’s rho = 0.428; *p* = 0.002; Figure 5B). In postoperative ICU patients, a moderate negative correlation was identified between calprotectin and PCT (Spearman’s rho = −0.48; *p* < 0.001; Figure 5C). No significant correlations were observed between calprotectin and other biomarkers in the non-surgical patient group. The Charlson comorbidity index (CCI) was calculated to describe comorbidity burden, with a median of 6 (IQR 4–9). However, CCI was not significantly associated with calprotectin levels or mortality in exploratory analyses and therefore was not included in the final regression models.

### Mortality analyses

Of the 124 patients included in the study, 38 died during their ICU stay, corresponding to an ICU mortality rate of 30.6%. Mortality analyses in this study were limited to the ICU period; no in-hospital or post-discharge mortality data were collected.

Higher SOFA and APACHE II scores, as well as elevated CRP levels, were significantly associated with mortality in the overall patient population (*p* < 0.01 for each). PCT showed borderline significance (*p* = 0.029), whereas calprotectin (*p* = 0.825) and lactate (*p* = 0.213) levels were not significantly associated with mortality (Table 2).

Similarly, in the sepsis subgroup, only SOFA, APACHE II, and PCT levels showed significant differences between survivors and non-survivors. Calprotectin, CRP, and lactate levels did not differ significantly between these groups (Table 2).

## Discussion

This study found no significant association between calprotectin levels and the presence of sepsis or mortality in patients in the surgical intensive care unit (ICU). Calprotectin levels were similar between patients with and without sepsis, and likewise, no significant differences were observed between survivors and non-survivors. These findings suggest that calprotectin may not be a sufficient standalone biomarker for diagnosing sepsis or predicting short-term mortality.

However, numerous studies in the literature have reported significantly elevated calprotectin levels during sepsis, with correlations to disease severity. In a 2024 study by Bohn et al. (8) investigating febrile infants, calprotectin demonstrated good diagnostic accuracy for bacterial infection, including sepsis, and performed comparably to PCT and CRP. Similarly, Larsson et al. (9) reported that calprotectin was superior to procalcitonin in predicting 30-day survival.

On the other hand, some studies emphasize the inconsistency of calprotectin’s prognostic value. Large-scale analyses in critically ill populations have suggested that calprotectin may not be as robust as CRP for sepsis diagnosis and offers limited utility in clinical decision-making when used alone (12). Our findings should be interpreted within the context of this conflicting body of evidence: calprotectin may not be a reliable indicator of sepsis or mortality across all clinical settings. In particular, in the surgical ICU—where postoperative inflammation is frequent and the patient population is heterogeneous—calprotectin did not demonstrate the diagnostic performance reported in some previous studies. This may be attributable to patient characteristics, sample size, or study design.

A notable finding in our study was the significant correlation between calprotectin and procalcitonin levels in patients diagnosed with sepsis and those in the postoperative monitoring group. This suggests that calprotectin may exhibit a biological response similar to PCT, particularly in inflammation driven by bacterial infection (8). Similarly, a study by Parke et al. (13) found that calprotectin had greater accuracy than PCT in predicting the need for mechanical ventilation and ICU admission among patients evaluated in the emergency department with suspected sepsis.

Our study found no significant correlations between calprotectin levels and SOFA or APACHE II scores. This suggests that calprotectin may not be directly associated with the severity of illness. Indeed, calprotectin levels showed a wide distribution across both high- and low-score groups. Similar findings have been reported in the literature, where no consistent association was observed between calprotectin and severity scores (14). Our results suggest that while calprotectin is sensitive to systemic inflammatory responses, its relationship with complex clinical outcomes such as organ dysfunction may be limited.

Interestingly, a negative correlation was found between calprotectin and CRP in patients requiring mechanical ventilation, possibly reflecting the complex nature of the inflammatory response in severe respiratory failure. Of note, among the 42 mechanically ventilated patients, 20 (47.6%) had sepsis while 22 (52.4%) were ventilated due to non-septic causes such as postoperative respiratory failure or underlying pulmonary disease. This heterogeneity may have influenced the observed association between calprotectin and CRP, as septic and non-septic respiratory failure likely involve different inflammatory pathways. These findings suggest that the relationship between calprotectin and PCT or CRP may vary depending on the clinical context, such as bacterial infection, respiratory failure, or postoperative inflammation. While CRP tends to rise in later phases, calprotectin reflects early neutrophil activation. This finding aligns with reports of elevated calprotectin levels in severe pulmonary infections (3). However, in our study, the association was observed in patients with respiratory failure requiring mechanical ventilation, which may reflect a broader spectrum of inflammatory pathways beyond infection alone. As this finding is based on subgroup analysis, its clinical relevance should be interpreted cautiously and confirmed by studies with larger sample sizes.

The data obtained indicate that calprotectin offers limited diagnostic and prognostic value compared to commonly used biomarkers such as CRP and PCT. In the postoperative and heterogeneous environment of a surgical ICU, calprotectin did not reliably predict sepsis or mortality. In contrast, CRP, PCT, and SOFA/APACHE II scores demonstrated more consistent and clinically relevant prognostic value for patient management. In our study, CRP levels and severity scores were significantly associated with mortality, whereas calprotectin did not provide any additional prognostic value. In the context of our surgical ICU population, calprotectin did not demonstrate sufficient prognostic value, and our findings are in line with other inconclusive studies. Therefore, while calprotectin may have potential in specific settings, current evidence does not yet support its use as a standardized prognostic tool in intensive care practice.

Nevertheless, the potential clinical relevance of calprotectin in specific patient subgroups should not be overlooked. The observed correlation between calprotectin and PCT in patients diagnosed with sepsis suggests that this biomarker may reflect infectious burden in bacterial infections. In cases of suspected infection, calprotectin may provide complementary information on the presence and severity of infection when evaluated in conjunction with PCT. Additionally, changes in calprotectin levels in patients who required mechanical ventilation suggest that this marker may have clinical value in acute inflammatory processes accompanied by widespread neutrophil activation (e.g., ARDS). Due to its rapid rise, calprotectin may offer earlier response detection than CRP, potentially providing theoretical advantages in differentiating early infections or monitoring inflammation. However, our study demonstrates that these potential benefits were not evident in a surgical ICU population. Therefore, broader prospective studies are needed to clarify the clinical utility of calprotectin. In light of current data, calprotectin is more appropriately evaluated as a complementary biomarker alongside others rather than as a comprehensive diagnostic or prognostic tool. More broadly, this limitation applies to most single biomarkers for sepsis, given the condition’s complexity and heterogeneity.

### Study limitations

This study has several limitations. First, the relatively small sample size limits the statistical power and generalizability of the findings. The insufficient number of patients in specific subgroups—such as those with postoperative status or sepsis—has made these subgroup analyses more challenging to interpret. Additionally, the heterogeneity of the included patient population, particularly the potential impact of non-septic inflammatory processes (e.g., surgical trauma, postoperative stress) on calprotectin levels, may have masked specific clinical patterns.

Another limitation is that the study was conducted at a single centre. This restricts the generalizability of the findings to surgical intensive care units with different patient populations. Furthermore, calprotectin measurements were performed only once and in the early phase of ICU admission. However, some studies have demonstrated that changes in calprotectin levels over time (e.g., within 24–72 h) may provide better predictive value for clinical course and mortality (8). In the present study, data on the dynamic follow-up of calprotectin were unavailable.

Additionally, both the technical characteristics of diagnostic assays—such as detection range, specificity, and detection limits—and pre-analytic factors may influence the comparability of results and overall test performance (14). Variations observed across studies, reflecting heterogeneity due to differences in cutoff values, further support this concern (7).

Future studies should consider prospective designs that include time-dependent changes in calprotectin levels, subgroup analyses supported by larger sample sizes, and multicenter comparative investigations. Moreover, it is essential to assess whether combining calprotectin with other biomarkers, such as CRP, PCT, and lactate, could enhance its diagnostic and prognostic utility (7, 10, 15, 16).

Another limitation of this study is the lack of precise data regarding the time elapsed since sepsis onset before ICU admission. In addition, although the Charlson comorbidity index was assessed to quantify comorbidity burden, it was not incorporated into the final regression models. This may have limited adjustment for comorbidities as potential confounders. While biomarker samples were collected within the first 24 h of ICU stay, the inflammatory status of patients may have varied depending on how early or late they were in the sepsis course. This temporal heterogeneity could have influenced calprotectin levels and reduced the accuracy of inter-patient comparisons.

## Conclusion

This study demonstrated that calprotectin levels alone are insufficient as a biomarker for sepsis diagnosis or short-term mortality prediction in surgical ICU patients. Its diagnostic and prognostic contribution was limited in the overall patient population. The inverse relationship between calprotectin and CRP in patients requiring mechanical ventilation is noteworthy. Additionally, the correlation observed with PCT in specific subgroups suggests that calprotectin may be a complementary indicator of inflammatory responses. Nonetheless, these findings must be supported by studies involving dynamic monitoring and more homogeneous patient groups.

## Data Availability

The raw data supporting the conclusions of this article will be made available by the authors, without undue reservation.
