# Supplementary material for: Is calprotectin a reliable marker in surgical ICU settings? A clinical evaluation of its role in sepsis and mortality prediction
Source: Front Med (Lausanne). 2025 Oct 10;12:1619825. doi: 10.3389/fmed.2025.1619825 (PMC12549553; doi:10.3389/fmed.2025.1619825)
Supplement: Supplementary file 1 [file Data_Sheet_1.docx]

**Calprotectin Measurement**

Based on the double-antibody sandwich principle, serum calprotectin levels were quantitatively measured using an ELISA kit from SunRed Bio (Shanghai, China). Venous blood samples were collected in dry tubes and centrifuged at 3000 rpm for 20 minutes; the resulting serum was stored at −80°C until analysis. During the assay, 40 μL of serum, 10 μL of CALPRO antibody, and 50 μL of Streptavidin-HRP were added to each well. The plate was sealed and incubated at 37°C for 60 minutes. The wells were then washed three times with a wash solution diluted at a 1:30 ratio. Subsequently, 50 μL of Chromogen A and 50 μL of Chromogen B were added to each well, and the mixture was incubated in the dark at 37°C for 10 minutes. The reaction was terminated with 50 μL of stop solution, and the optical density was measured at 450 nm. Calprotectin levels were calculated from the standard curve and expressed in nanograms per milliliter (ng/mL). The assay range was 0.15–40 ng/mL; intra-assay variation was <10% and inter-assay variation was <12%.
